# Similar and Additive Effects of Ovariectomy and Diabetes on Insulin Resistance and Lipid Metabolism

**DOI:** 10.1155/2015/567945

**Published:** 2015-03-05

**Authors:** Shady H. Tawfik, Bothaina F. Mahmoud, Mohamed I. Saad, Mona Shehata, Maher A. Kamel, Madiha H. Helmy

**Affiliations:** ^1^Department of Biochemistry, Medical Research Institute, Alexandria University, Egypt; ^2^Department of Physiology, Medical Research Institute, Alexandria University, Egypt

## Abstract

Type 2 diabetes mellitus (T2DM) is among the leading causes of death in postmenopausal women. The disruption of ovarian function may contribute to the incidence of T2DM. The purpose of this study was to investigate the effects of ovariectomy and T2DM on glucose and lipid homeostasis, perilipin levels in adipose tissues, as a lipolytic regulator, and levels of certain adipokines. Ovariectomized (OVX) rats were used as a model for postmenopausal women. The study was performed on sham, OVX, sham diabetic, and OVX diabetic female rats. The results indicated that ovariectomy alters adipose tissue metabolism through reducing perilipin content in white adipose tissue (WAT); however it has no effect on perilipin level in brown adipose tissue (BAT). OVX diabetic females suffer from serious metabolic disturbances, suggested by exacerbation of insulin resistance in terms of disrupted lipid profile, higher HOMA-IR, hyperinsulinemia, higher leptin, and lower adiponectin concentrations. These metabolic derangements may underlie the predisposition for cardiovascular disease in women after menopause. Therefore, for efficient treatment, the menopausal status of diabetic female should be addressed, and the order of events is of great importance because ovariectomy following development of diabetes has more serious complications compared to development of diabetes as result of menopause.

## 1. Introduction

Type 2 diabetes mellitus (T2DM) is a group of metabolic disorders of fuel homeostasis manifested mainly by hyperglycemia and dysregulated lipid metabolism. The metabolic defects that underlie the development of T2DM are islet *β* cells dysfunction, hyperglucagonemia, elevated endogenous glucose production, development of peripheral insulin resistance, inflammation of adipose tissue, and dysregulation of adipokines production [1].

Adipose tissues are either white or brown [2]. The primary function of white adipose tissue (WAT) is to store excess fats in the form of triglycerides, whereas regulation of nonshivering thermogenesis through uncoupling protein-1 (UCP-1) is the main function of brown adipose tissue (BAT) [3]. Phosphorylated hormone sensitive lipase (HSL), the key enzyme regulating lipolysis, hydrolyzes triglycerides to nonesterified fatty acids (NEFA) and glycerol. Perilipin, a critical lipolytic regulator in adipocytes, is a protein which covers lipid droplet surfaces and acts as a physical barrier, protecting stored triglyceride from hydrolysis by cellular lipases. Under lipolytic conditions, phosphorylated perilipin dissociates from the droplet surface and enhances HSL accessibility to lipid droplets [4]. Perilipin-null mice show constitutive activation of basal lipolysis and attenuation of hormone-induced lipolysis and are characterized by a remarkable reduction in adipose tissue, increased food intake, and energy expenditure; however, they develop insulin resistance [5].

It is widely appreciated that adipose tissue acts as an active endocrine organ which secrets a wide range of protein factors referred to as adipokines [6]. Adipokines, for example, adiponectin and leptin, play crucial roles in multiple processes such as inflammation, metabolism, insulin resistance, and obesity-related conditions [6]. Decreased adiponectin levels have been associated with obesity, insulin resistance, coronary heart disease, and nonalcoholic fatty liver disease (NAFLD). Moreover, adiponectin has anti-inflammatory, antithrombotic, and antiatherogenic actions [6]. Leptin acts as a metabolic adipostat by repressing appetite and promoting energy expenditure. High circulating leptin levels have been associated with obesity and insulin resistance, reflecting a state of leptin resistance [7].

Estrogens play key protective roles against the development of obesity and metabolic diseases. Estrogens are important regulators of several metabolic processes, including glucose and lipid metabolism, body weight, adipose tissue distribution, caloric intake, and energy expenditure in both males and females [8].

Excess visceral (abdominal) adipose tissue is generally associated with adipocyte dysfunction and inflammation which could lead to systemic insulin resistance and metabolic syndrome. T2DM and cardiovascular disease are among the leading causes of death in postmenopausal women, and their prevalence in women after menopause suggests that the disruption of ovarian function may contribute to the incidence of these conditions [9]. Therefore, shedding light on the similarities and differences in metabolic disturbances occurs during T2DM; ovarian dysfunction and the combined effect of both conditions would be of basic and potential clinical importance.

The purpose of this study was to investigate the effects of female sex steroid deficiency and T2DM on glucose and lipid homeostasis, perilipin levels in adipose tissues, and production of certain adipokines (e.g., adiponectin and leptin). Our study was performed on sham-operated control, ovariectomized (OVX), sham-operated diabetic, and OVX diabetic female rats. The latter was used as a model for diabetic women in postmenopausal stage.

## 2. Materials and Methods

### 2.1. Experimental Animals

Female Wistar rats aged three months and weighing 180–200 g were obtained from the Medical Research Institute (Alexandria, Egypt). The rats were housed 4 per cage at an ambient temperature of 23 ± 1°C with 12/12 h light/dark cycles and 45 ± 5% humidity. Rats had free access to chow diet and water for a week before the study.

### 2.2. Induction of Diabetes

T2DM was induced in rats by single dose intraperitoneal injection of streptozotocin (STZ) (100 mg/kg dissolved in 0.05 M citrate buffer, pH 4.5, immediately before use) at the 5th day of birth. Confirmation of diabetes was done by presence of mild hyperglycemia (fasting blood glucose level > 150 mg/dL) after 8 weeks of STZ injection [10].

### 2.3. Experimental Design

The experimental animals were randomly divided into four groups, each group consisting of 10 rats detailed as follows.* Group (1)* served as the sham control rats; after being anesthetized, rats had sham surgical procedure (but not ovariectomy) and were allowed to recover for 4 weeks.* Group (2)* served as the ovariectomized (OVX) control rats; after being anesthetized, rats were bilaterally ovariectomized through midline incision (a procedure which is frequently used to disturb the signalling axis of female sex hormones [11]) and allowed to recover for 4 weeks.* Group (3)* served as the diabetic rats which had undergone sham surgical procedure and allowed to recover for 4 weeks.* Group (4)* served as the OVX diabetic rats; diabetic rats were anesthetized, bilaterally ovariectomized through midline incision, and allowed to recover for 4 weeks. After recovery, rats were kept in their standard cages for 8 weeks with free access to chow diet and water. Thereafter, the rats were fasted overnight, anaesthetized, and sacrificed by cervical decapitation. The blood was collected for serum separation and biochemical analysis. The liver and adipose tissues (white and brown) were excised immediately and stored at −80°C. White and brown adipose tissues (WAT and BAT) were obtained from visceral (retroperitoneal) and interscapular adipose depots, respectively.

### 2.4. Determination of Glucose, Insulin, and Lipid Profile

The level of serum glucose was estimated using an Accu-Chek Active glucometer. Serum insulin level was assayed using a sandwich ELISA kit (Millipore) according to the manufacturer's instructions.

Lipid profile was assessed by using a commercial diagnostic kit (Randox (UK)) according to the manufacturer's instructions.

### 2.5. Determination of Homeostasis Model of Insulin Resistance (HOMA-IR)

The insulin resistance index (IRI) was assessed by homeostasis model assessment estimate of insulin resistance (HOMA-IR) as follows:

(1) IRI=Fasting  insulin  μIU/mL×Fasting  glucose  mmol/L22.5.

### 2.6. Assay of Hepatic Lipid Content

About 2 g of liver was homogenized, and lipids were extracted with a chloroform:methanol mixture (2 : 1 v/v) as described by Folch et al. [12]. The concentration of liver cholesterol and triglycerides in the lipid extracts was measured enzymatically by using a kit (Randox (UK)) according to the manufacturer's instructions.

### 2.7. Determination of Perilipin Levels in Adipose Tissues

Levels of perilipin in rat adipose tissues were assessed using an ELISA kit (CUSABIO, China) according to the manufacturer's instructions. Perilipin level was calculated in terms of protein content in each tissue sample measured by the modified Lowry et al. method [13] for total protein determination.

### 2.8. Assay of Serum NEFA, Adiponectin, and Leptin Levels

Levels of NEFA, adiponectin, and leptin in rat serum were assessed using ELISA kits (MyBioSource, Chemicon, and RayBio, resp.) according to the manufacturer's instructions.

### 2.9. Statistical Analysis

The data were analyzed using the one-way analysis of variance (ANOVA) followed by LSD test to compare different groups with each other (SPSS software). Results were expressed as mean ± standard deviation (SD) and values of *p* > 0.05 were considered nonsignificantly different, while those of *p* < 0.05 were considered significant.

## 3. Results

### 3.1. Glucose Homeostasis Parameters

OVX control, diabetic, and OVX diabetic rats showed a significant increase in fasting blood glucose (FBG) level when compared to sham group. Moreover, FBG level was significantly increased in OVX diabetic rats compared to OVX control group. Although OVX diabetic group showed the highest rise in FBG level, this elevation was not significantly different from sham diabetic group (Table 1).

Insulin levels were significantly increased in OVX control, diabetic, and OVX diabetic rats when compared to sham group. Furthermore, OVX diabetic rats showed a significant increase in insulin levels when compared to OVX control and sham diabetic groups (Table 1).

The insulin resistance index calculated by the HOMA model (HOMA-IR) using level of fasting insulin (*μ*IU/mL) and glucose level (mmol/L) indicated that OVX control, diabetic, and OVX diabetic groups exhibited higher HOMA-IR values when compared to sham group. Furthermore, OVX diabetic rats showed a significant increase in HOMA-IR value when compared to both OVX control and sham diabetic groups (Table 1).

### 3.2. Lipid Profile and Hepatic Lipid Content

OVX control, diabetic, and OVX diabetic rats showed a significant increase in serum and hepatic triglycerides levels when compared to sham group. Furthermore, serum and hepatic triglycerides levels of OVX diabetic rats were significantly elevated when compared to OVX control and sham diabetic groups. Hepatic cholesterol content of OVX control, diabetic, and OVX diabetic rats showed a significant rise compared to sham group (Table 1).

OVX control, diabetic, and OVX diabetic rats showed a significant increase in serum total cholesterol, LDL-cholesterol, and NEFA when compared to sham group. Moreover, these parameters were significantly increased in OVX diabetic rats when compared to OVX control and sham diabetic groups. Also, these parameters were significantly elevated in sham diabetic group compared to OVX control group (Table 1).

OVX control, diabetic, and OVX diabetic rats showed a significant reduction in serum HDL-cholesterol compared to sham group. HDL-cholesterol level was significantly decreased in OVX diabetic rats when compared to OVX control and sham diabetic groups. Also, sham diabetic group showed a significant decrease in HDL-cholesterol compared to OVX control group (Table 1).

### 3.3. Perilipin Level in Adipose Tissues

**Figure 1:**
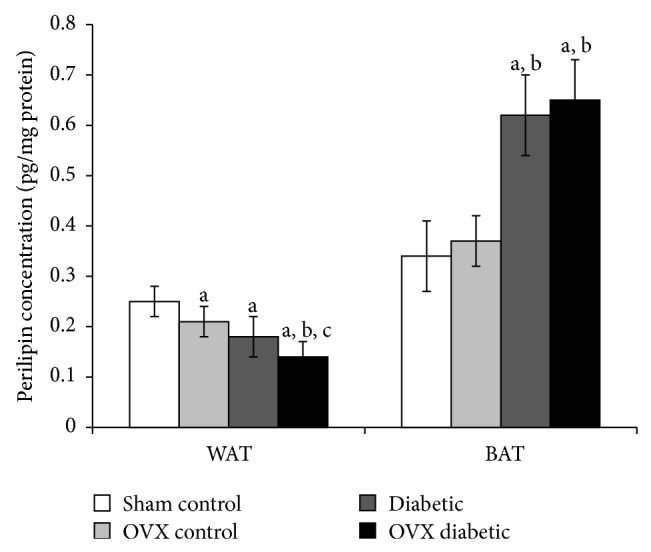
Perilipin level (pg/mg protein) in white and brown adipose tissues of different studied groups at the end of the study. The abbreviations denote the following: Sham: sham-operated rats; OVX: ovariectomized rats; WAT: white adipose tissue; BAT: brown adipose tissue. a: significantly different from the sham control group, b: significantly different from the OVX control group, and c: significantly different from the diabetic group, using ANOVA (LSD), *P* value < 0.05.

In WAT, perilipin levels of OVX control, diabetic, and OVX diabetic rats (0.21 ± 0.03, 0.18 ± 0.04, and 0.14 ± 0.03 pg/mg protein, resp.) were significantly reduced compared to sham group (0.25 ± 0.03 pg/mg protein). Also, perilipin level of OVX diabetic group was significantly decreased from the perilipin level of both OVX control and diabetic groups (Figure 1).

In BAT, ovariectomy per se did not cause a significantly different change in perilipin level compared to the sham group. Moreover, diabetic and OVX diabetic groups (0.62 ± 0.08 and 0.65 ± 0.08 pg/mg protein, resp.) demonstrated a significantly elevated concentration of perilipin to be 1.8- and 1.9-fold higher than the sham group and 1.6- and 1.7-fold higher than the OVX group, respectively (Figure 1).

### 3.4. Levels of Adipokines in Serum and Adipose Tissues

**Figure 2:**
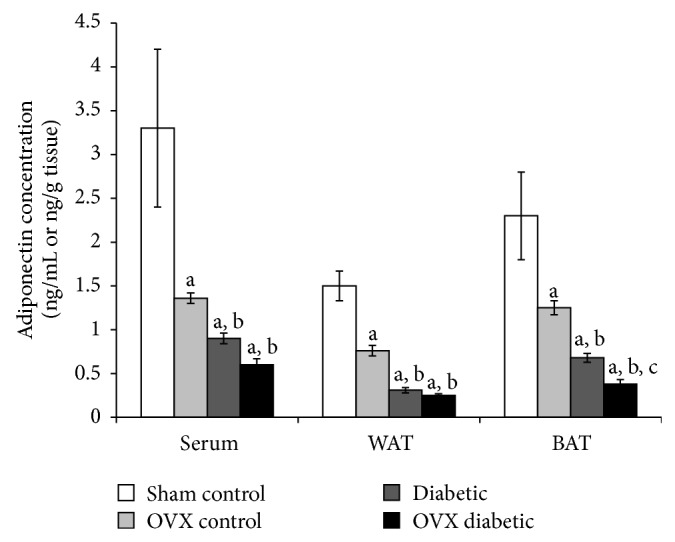
Adiponectin level in serum (ng/mL) and adipose tissues (ng/g tissue) of different studied groups at the end of the study. The abbreviations denote the following: Sham: sham-operated rats; OVX: ovariectomized rats; WAT: white adipose tissue; BAT: brown adipose tissue. a: significantly different from the sham control group, b: significantly different from the OVX control group, and c: significantly different from the diabetic group, using ANOVA (LSD), *P* value < 0.05.

Serum adiponectin levels of OVX control, diabetic, and OVX diabetic groups were significantly reduced to be 0.4-, 0.27-, and 0.18-fold lower than the sham group, respectively. Also, serum adiponectin levels of diabetic and OVX diabetic groups (0.9 ± 0.06 and 0.6 ± 0.07 ng/mL, resp.) were significantly decreased compared to OVX control group (1.36 ± 0.06 ng/mL) (Figure 2). WAT and BAT levels exhibited quite similar pattern of change to that of serum adiponectin level. In WAT, adiponectin levels of OVX control, diabetic, and OVX diabetic groups were 0.5-, 0.2-, and 0.16-fold lower than the sham group, respectively, whereas, in BAT, adiponectin levels were 0.5-, 0.3-, and 0.16-fold lower than the sham group, respectively. In addition, adiponectin level of OVX diabetic group in BAT (0.38 ± 0.05 ng/g tissue) was significantly the lowest level compared to other studied groups (Figure 2).

**Figure 3:**
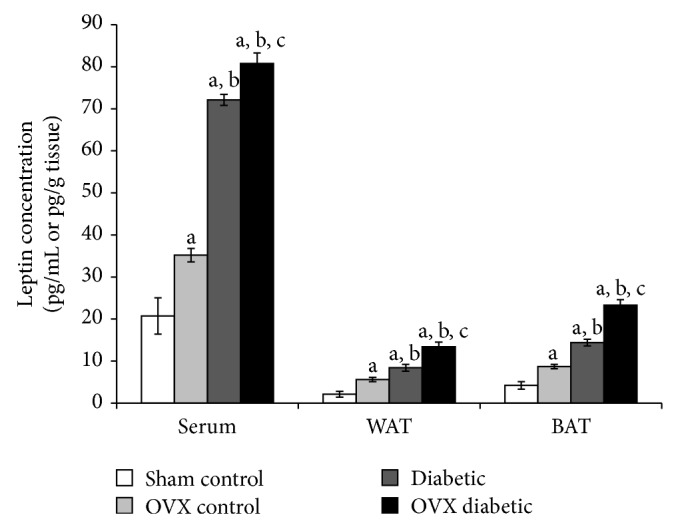
Leptin level in serum (pg/mL) and adipose tissues (pg/g tissue) of different studied groups at the end of the study. The abbreviations denote the following: Sham: sham-operated rats; OVX: ovariectomized rats; WAT: white adipose tissue; BAT: brown adipose tissue. a: significantly different from the sham control group, b: significantly different from the OVX control group, and c: significantly different from the diabetic group, using ANOVA (LSD), *P* value < 0.05.

On the contrary, serum leptin levels of OVX control, diabetic, and OVX diabetic groups were significantly increased to be 1.7-, 3.5-, and 3.9-fold higher than the sham group, respectively. Also, serum leptin level of diabetic and OVX diabetic groups (72.01 ± 1.3 and 80.8 ± 2.5 pg/mL, resp.) was significantly elevated compared to OVX control group (35.2 ± 1.6 pg/mL). In addition, serum leptin level of OVX diabetic group was significantly the highest level compared to other studied groups (Figure 3). WAT and BAT leptin levels exhibited similar patterns of change. In WAT, leptin levels of OVX control, diabetic, and OVX diabetic groups were 2.6-, 4-, and 6.3-fold higher than the sham group, respectively, while, in BAT, leptin levels were 2-, 3.4-, and 5.5-fold higher than the sham group, respectively (Figure 3).

## 4. Discussion

The role of sex difference and gonadal hormones in modulating insulin sensitivity and glucose tolerance is of great interest owing to the increasing prevalence of insulin resistance and its associated disorders such as T2DM, metabolic syndrome, and obesity, which have adverse effects on women's health from pregnancy to fetus to menopausal woman [14]. In females with normal ovarian function, lipid storage in subcutaneous adipose tissue (gynoid pattern) is metabolically favored over visceral lipid storage (android type) [15]. However, disruption of female sex steroid function, such as following menopause, hysterectomy, or breast cancer treatment, is associated with excess visceral adiposity [16]. For instance, the risk of developing metabolic syndrome in women after menopause is 60%, whereas in age-matched women before menopause the risk is only 20–30%. However, little is known regarding how ovarian hormones affect the metabolic function of adipose tissue [11]. Therefore, the metabolic effect of T2DM and ovarian hormone deficiency on adipose tissue metabolism and distribution would be of basic and potential clinical importance.

The present study aimed to investigate the mutual effects of ovarian hormone deficiency and T2DM on glucose and lipid homeostasis, adipose tissues levels of perilipin, as a critical regulator of lipolysis, and levels of certain adipokines in serum and adipose tissues.

The present study showed that the ovariectomy-induced disturbed metabolism is associated with dysregulated glucose homeostasis; OVX control group showed significant elevation of FBG and insulin levels compared to sham control rats; however the FBG level was still within the normal range. This could be explained by the fact that deficiency of female sex hormones results in declined insulin-stimulated glucose disposal [17]. The diabetic females, which already had hyperglycemia, showed nonsignificant elevation in FBG after ovariectomy. Regarding HOMA-IR, our results indicated that both ovariectomy and diabetes caused insulin resistance (diabetes caused higher HOMA-IR level), and the combined effect of both conditions resulted in exacerbation of insulin resistance state in female rats.

Insulin sensitivity is the ability of insulin to lower FBG by suppressing hepatic glucose production and promoting glucose uptake in peripheral tissues. Insulin resistance is defined as an impaired biological response to insulin; however there is sufficient variability in normal sensitivity to insulin that there is no distinct value at which resistance starts and sensitivity ends [18]. Therefore, there is no absolute definition of hyperinsulinemia, since an insulin level that is raised for an individual is usually still within the wide range of normality [19].

Several clinical and experimental studies suggested that there is metabolic interaction between insulin and female sex hormones; for example, the low levels of female sex steroids associated with menopause are related to a decline in whole body insulin-mediated glucose uptake. However, the exact cellular mechanisms behind this state of insulin resistance and the role of low female sex steroids are not fully understood [20]. Therefore, the decline in ovarian steroids function could result in alterations in circulating insulin levels and/or insulin receptor sensitivity.

These disturbances of glucose homeostasis after ovariectomy were associated with serious derangements in lipid profile as indicated by a significant increase in serum total cholesterol, triglycerides, LDL-cholesterol, and NEFA as well as a significant decrease in HDL-cholesterol. OVX rats had milder disturbance in serum lipids than diabetic rats, but rats that had both conditions showed more serious disruption in serum lipids. In addition, the present study indicated that the hepatic triglycerides increased significantly as a result of ovariectomy. OVX control rats exhibited 8% elevation of hepatic triglycerides, while OVX diabetic rats showed 25% increase in the hepatic triglycerides which may imply that the diabetic females following ovariectomy (or following menopause) are at more risk for the development of NAFLD than OVX or diabetic females. The observed derangements in serum lipids may be a cause or a consequence of insulin resistance [21].

From the results of our study, it was clear that the adipose tissue contents of perilipin are differentially regulated depending on the type of adipose tissue (white or brown) and the type of insult (ovariectomy, diabetes, or both). Perilipin level in WAT was significantly declined in the OVX females compared to normal females, while its level in BAT showed no significant change as a result of ovariectomy. Diabetic females showed a similar extent of decline in WAT-perilipin level as that observed in OVX rats. On the other hand, BAT perilipin level was significantly elevated (about 2-fold higher than the control value) in diabetic females. In OVX diabetic rats, the WAT-perilipin showed further significant decline compared to OVX females and diabetic females, while BAT-perilipin showed no significant change compared to diabetic females. These patterns of change indicated that WAT-perilipin significantly decreased by ovariectomy and T2DM and when both conditions were combined, there was cumulative or synergistic effect; however, the BAT-perilipin level appeared to be enhanced by diabetic state but not affected by the ovariectomy of normal or diabetic females. These results might imply that WAT, but not BAT, perilipin protein level is under direct regulation by ovarian hormones.

In lipolytically active adipocytes, elevated intracellular cyclic adenosine monophosphate (cAMP) levels following hormonal stimulation result in phosphorylation of perilipin which rapidly initiates lipolysis of triglycerides to glycerol and fatty acids by phosphorylated HSL. Previously, our lab demonstrated that the diabetic state differentially affects cAMP levels according to the type of adipose tissue; WAT showed increased cAMP level, while BAT showed a decline in cAMP level [22]. Moreover, unrestrained lipolysis increases circulating levels of NEFA in the absorptive state, the driving force of insulin resistance. The increased flux of NEFA affects insulin signalling, reduces glucose uptake in muscle, exaggerates triglyceride synthesis, induces glucose production in the liver, and contributes to *β*-cell failure [23].

From the above studies, it was suggested that the low content of perilipin is associated with enhanced lipolysis. The observed low level of perilipin in WAT of OVX and diabetic rats could be explained by insulin resistance state which favors the lipolytic pathway as a result of increased glucagon/insulin ratio and the produced NEFA and glycerol may further exaggerate insulin resistance in a vicious cycle. Consistently, the results indicated elevated serum level of NEFA in OVX and diabetic rats.

On the other hand, BAT of diabetic rats showed elevated perilipin which could be explained by the fact that BAT is more insulin sensitive than WAT [24] and may retain insulin sensitivity even under insulin resistance. This declined cAMP and elevated perilipin levels in BAT may result in shifting in its metabolic capacity from lipolytic and catabolic into lipogenic and anabolic pathways which could change cells phenotype from BAT (metabolically active) into WAT (storage) phenotype [22].

Consistent with our results, both human and animal studies suggested associations between perilipin expression and adiposity, lipid metabolism, and glucose homeostasis [25]. For example, perilipin-null mice show constitutive activation of basal lipolysis and attenuation of hormone-induced lipolysis and are characterized by a remarkable reduction in adipose tissue, increased food intake, and energy expenditure; however, they develop insulin resistance [5]. Moreover, it was demonstrated that OVX mice exhibit increased serum glycerol and NEFA levels and express significantly lower perilipin protein content in WAT [26].

Adipose tissue dysfunction causes disrupted production or secretion of adipokines that could contribute to the pathogenesis of insulin resistance and obesity-related complications. For example, obesity, insulin resistance, and T2DM are associated with elevated level of leptin and decreased level of adiponectin, reflecting a state of leptin resistance and adiponectin deficiency [2, 6]. In line with this, our results indicated that OVX and diabetic female rats developed insulin resistance, suggested by the elevated level of leptin and reduced level of adiponectin in serum and adipose tissues. Moreover, ovariectomy and diabetes together exaggerated this state of insulin resistance in terms of leptin resistance and adiponectin deficiency.

Although the relation between metabolic diseases and certain adipokines is well-established, the association between adipokines level and endogenous and exogenous sex hormones or menopausal stage is highly controversial and inconclusive. It was reported that estrogens stimulate the production of leptin from adipose cells from women, but not from men [27]. Various studies have shown differences in leptin values in pre- and postmenopausal women; however, the existing clinical data are discordant [28]. For instance, Hadji et al. demonstrated that serum leptin levels were significantly higher in both pre- and postmenopausal obese women, and leptin levels were not influenced by menopausal status or serum estradiol level [29]. Moreover, while hormone replacement therapy (HRT) caused an elevation of the plasma leptin levels in postmenopausal women as indicated by Konukoglu et al. [30], low HRT dose was associated with a reduction in serum leptin, at least in young, recently menopause women [31]. Surprisingly, Lambrinoudaki et al. demonstrated that HRT did not exert any effect on serum leptin in lean or overweight postmenopausal women [32].

Circulating adiponectin levels are higher in women than men, but the biological explanation for this sex difference is unknown. However, estrogen is thought to exert a negative, rather than a positive, influence on circulating adiponectin [33]. Consistently, ovariectomized mice resulted in an increase in plasma adiponectin that is reversed by estradiol replacement therapy [34]. In contrast to this, aged estradiol-deficient animals (ovariectomized) exhibited decreased circulating adiponectin and reduced AdipoR2 levels [35]. Moreover, it has been shown that ovarian hormone deficiency leads to obesity and visceral adiposity in rats, but surprisingly it was not associated with change in adiponectin levels [36].

Furthermore, the influence of menopause on serum adiponectin was also inconclusive; while some studies showed no effect, others reported higher adiponectin levels in postmenopausal compared to premenopausal women after adjusting for age, fat mass, and fat distribution [33]. For example, in Japanese women, adiponectin concentrations were similar in premenopausal and postmenopausal women [37]. In contrast, obese Tunisian postmenopausal women showed increase of plasma adiponectin compared to premenopausal women [38].

Similarly, studies investigating serum adiponectin level during pregnancy are contradictory; while some studies reported no significant alterations in adiponectin concentrations during the 3 trimesters of pregnancy, others showed lower adiponectin levels. Moreover, hypoadiponectinemia can be associated with woman's health disorders such as infertility, gestational diabetes, polycystic ovary syndrome (PCO), endometriosis, and endometrial cancer [39]. Therefore, regardless of the well-established metabolic effects, adipokines concentration differs greatly in females and these differences depend on the level and the composition of endogenous sex hormones, adipose tissue integrity and distribution, the menopausal stage, presence of pregnancy, and other concurrent health problems.

## 5. Conclusion

In conclusion, it was clear that ovarian steroid imbalance induced by ovariectomy alters adipose tissue metabolism through reducing perilipin content in WAT; however it has no effects on the BAT perilipin level. It was also concluded that ovariectomized females (or postmenopausal) after being diabetic suffer from more serious disturbances in lipid and carbohydrate metabolism, suggested by exacerbation of insulin resistance state in terms of disrupted lipid profile, higher HOMA-IR, hyperinsulinemia, higher leptin, and lower adiponectin concentrations. These metabolic derangements may underlie the predisposition for cardiovascular disease and NAFLD in women after menopause.

Therefore, for efficient selection and adjustment of the treatment, the menopausal status of diabetic female should be addressed and the order of events is of great importance because ovariectomy following the development of diabetes has more serious complications compared to the development of diabetes as result of ovariectomy (or following menopause).

## Figures and Tables

**Table 1:** Parameters of glucose homeostasis, lipid profile, and hepatic lipid content of different studied groups at the end of the study.

Parameter	Sham control	OVX control	Diabetic	OVX diabetic
Fasting blood glucose (mg/dl)	86.9 ± 11.8	104.1 ± 5.9^a^	159.7 ± 8.3^a,b^	174.4 ± 30.7^a,b^
Serum insulin (*μ*IU/ml)	4.8 ± 1.8	13.4 ± 1.1^a^	22.1 ± 1.0^a,b^	29.3 ± 1.5^a,b,c^
HOMA-IR	0.96 ± 0.3	3.45 ± 0.3^a^	8.67 ± 0.5^a,b^	12.6 ± 2.2^a,b,c^

Triglycerides (mg/dl)	89.9 ± 9.8	115.2 ± 5.4^a^	126.8 ± 4.73^a^	142.8 ± 26.2^a,b,c^
Total cholesterol (mg/dl)	143.6 ± 21.3	194.5 ± 6.5^a^	213.5 ± 3.6^a,b^	241.4 ± 4.8^a,b,c^
LDL cholesterol (mg/dl)	75.5 ± 17.7	127.9 ± 6.4^a^	150.4 ± 4.6^a,b^	181.6 ± 9.4^a,b,c^
HDL cholesterol (mg/dl)	49.9 ± 6.3	43.5 ± 1.87^a^	37.8 ± 2.1^a,b^	31.1 ± 2.7^a,b,c^
Serum NEFA (mmol/dl)	5.7 ± 1.4	12.2 ± 1.1^a^	32.2 ± 1.5^a,b^	39.5 ± 2.1^a,b,c^

Hepatic triglycerides content (mg/g liver)	102.4 ± 8.5	110.6 ± 7.4^a^	130.1 ± 6.7^a,b^	163.7 ± 9.3^a,b,c^
Hepatic cholesterol content (mg/g liver)	40.9 ± 4.1	46.5 ± 2.2^a^	46.4 ± 3.1^a^	48.2 ± 4.9^a^

Values are presented as mean ± SD (*n* = 10). Sham: sham-operated rats, OVX: ovariectomized rats. ^a^Significantly different from the sham control group, ^b^significantly different from the OVX control group, and ^c^significantly different from the diabetic group, using ANOVA (LSD), *p* value < 0.05.

## Abbreviations

T2DM: Type 2 diabetes mellitus
NEFA: Nonesterified fatty acids
WAT: White adipose tissue
BAT: Brown adipose tissue
STZ: Streptozotocin
OVX: Ovariectomized
HSL: Hormone sensitive lipase
UCP-1: Uncoupling protein-1
FBG: Fasting blood glucose
NAFLD: Nonalcoholic fatty liver disease
IRI: Insulin resistance index
HOMA-IR: Homeostasis model assessment estimate of insulin resistance
ELISA: Enzyme-linked immunosorbent assay
ANOVA: Analysis of variance
cAMP: Cyclic adenosine monophosphate
FSH: Follicle stimulating hormone
PCO: Polycystic ovary syndrome
HRT: Hormone replacement therapy.
